# The National Centre for Gaming Disorders – the Demographic Profile and Clinical Characteristics of Individuals Accessing Our Service

**DOI:** 10.1192/bjo.2024.507

**Published:** 2024-08-01

**Authors:** Theodore Piper, Michelle Da Silva Youngs, Haby Tomy, Rebecca Lockwood, Henrietta Bowden-Jones

**Affiliations:** 1 The National Centre for Gaming Disorders, London, United Kingdom; 2 National Centre for Behavioural Addictions, London, United Kingdom; 3 University College London, London, United Kingdom; 4 University of Cambridge, Cambridge, United Kingdom

## Abstract

**Aims:**

Gaming Disorder (GD) was recognised in the addiction field by the International Statistical Classification of Diseases and Related Health Problems in 2018. The National Centre for Gaming Disorders (NCGD) is the first NHS clinic to accept referrals from adults and young people who are struggling with the characteristics of GD. The NCGD opened in 2019. Since then, we have received over 1,000 referrals from either gamers, their family members, or from parents seeking support. The team is multidisciplinary and led by Addiction Psychiatrists and Consultant Psychologists.

This service evaluation aims to understand the demographics, clinical characteristics, and gaming behaviours and trends of those with a GD who are accessing our service.

**Methods:**

The data included in this service evaluation is based on 380 gamer referrals. Data was collected through our referral form.

**Results:**

Demographics: The average age of gamers at referral was 19 years, with 60% of gamers aged between 13–18 years old. Male gamers represented 90% of the sample, with the remaining 10% made up of gamers identifying as female (9%), trans, or other. People who identify as White (British, Irish, or Other) represent 74% of referrals. The remaining 26% are from individuals who identify as Asian or Asian British, Mixed, Black or Black British, or of other ethnicities. Individuals based in London, or the South-East of England make up 60% of referrals. Comorbidities: 1 in 10 gamers had been formally diagnosed with a neurodevelopmental disorder at the time of referral. 1 in 8 gamers had an existing mental health condition. Gaming Trends: The three most popular games played were Fortnite, Minecraft, and Call of Duty. Our sample spent on average 10 hours per day gaming. In-game purchases were present in 17% of gamers. The average in-game expenditure at the point of referral was £4,500. In our sample, 46% were aggressive and 30% were physically violent to family members when interrupted from gaming.

**Conclusion:**

As of date, we have had 530 gamer referrals, and we are continuing to extract relevant information on the demographics and characteristics of individuals with a GD. Our data suggests that the typical gamer accessing our service is male, young, white, and from London. The most popular game played is Fortnite. A substantial proportion of our sample are aggressive or physically violent to family members when their gaming is interrupted, whilst others are at risk of spending thousands of pounds on in-game purchases.

